# Research on a Defects Detection Method in the Ferrite Phase Shifter Cementing Process Based on a Multi-Sensor Prognostic and Health Management (PHM) System

**DOI:** 10.3390/s16081263

**Published:** 2016-08-10

**Authors:** Bo Wan, Guicui Fu, Yanruoyue Li, Youhu Zhao

**Affiliations:** 1 School of Reliability and Systems Engineering, Beihang University, Beijing 100191, China; fuguicui@buaa.edu.cn (G.F.); 15652928449@163.com (Y.L.); 2 Dipartimento di Energia, Politecnico di Milano, Milano 20133, Italy; zyhdbh@sina.com

**Keywords:** multi-sensor, prognostic and health management, cementing strength

## Abstract

The cementing manufacturing process of ferrite phase shifters has the defect that cementing strength is insufficient and fractures always appear. A detection method of these defects was studied utilizing the multi-sensors Prognostic and Health Management (PHM) theory. Aiming at these process defects, the reasons that lead to defects are analyzed in this paper. In the meanwhile, the key process parameters were determined and Differential Scanning Calorimetry (DSC) tests during the cure process of resin cementing were carried out. At the same time, in order to get data on changing cementing strength, multiple-group cementing process tests of different key process parameters were designed and conducted. A relational model of cementing strength and cure temperature, time and pressure was established, by combining data of DSC and process tests as well as based on the Avrami formula. Through sensitivity analysis for three process parameters, the on-line detection decision criterion and the process parameters which have obvious impact on cementing strength were determined. A PHM system with multiple temperature and pressure sensors was established on this basis, and then, on-line detection, diagnosis and control for ferrite phase shifter cementing process defects were realized. It was verified by subsequent process that the on-line detection system improved the reliability of the ferrite phase shifter cementing process and reduced the incidence of insufficient cementing strength defects.

## 1. Introduction

**Figure 1:**
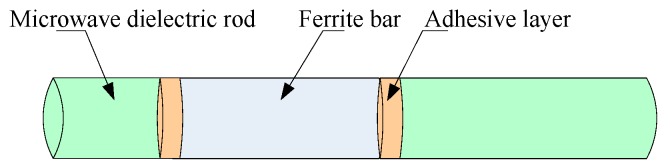
Brief diagram of the ferrite phase shifter cementing process flow.

The ferrite phase shifter is a primary unit of any radar apparatus. Cementing fractures are one of the failure phenomena that always appear during application [1]. As an important part of a ferrite phase shifter’s manufacture, the reliability of the cementing process influences the unit’s serviceability directly [2,3,4,5,6]. Figure 1 illustrates the cementing process of a ferrite phase shifter.

**Figure 2:**
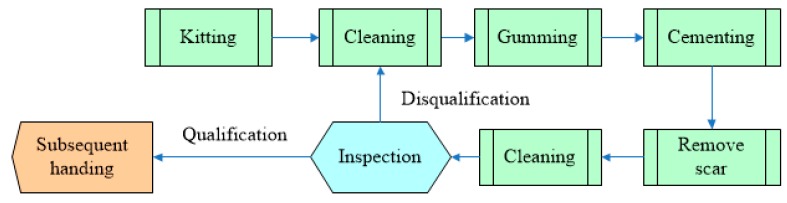
Cementing process flow of ferrite phase shifter.

The cementing process bonds the microwave dielectric rods 1 and 2 and the ferrite bar in order and must meet the requirements of size and strength. Its process flow is shown in Figure 2. As shown in Figure 2, the cementing process is as follows: (a) kitting: equipping all components, materials and instruments according to blueprints and schedules; (b) cleaning: scrubbing the glued region with cotton wool dipped in alcohol, and then preheating; (c) gumming: uniformly applying TSH-4 glue on the bonding surface of components; (d) cementing: putting the ferrite bar and microwave dielectric rod into the special tooling according to the position shown in the blueprint, applying axial and radial stress respectively to locate the components, and putting into the oven for curing; (e) removing scars: clamping the workpiece in the lathe and removing the excessive excess scarring of the adhesive surface; (f) cleaning: running water is used to clean the shifter; (g) inspection: dimension, coaxial degree and straightness of the bonding parts need to be inspected in this step.

**Figure 3:**
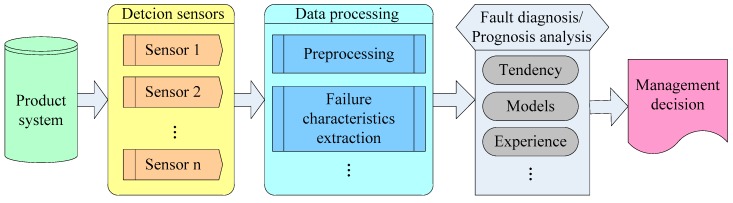
Multi-sensors PHM technology frame.

PHM technology is a method that used to evaluate the working state of a product or a system under actual application condition. The definition of “Health” is the product’s degradation or the degree of deviation, relative to the expected normal state. “Prognostic” means predicting future health condition according to history and current health state [7,8,9,10]. The core idea of PHM technology is researching system’s degradation or deviation under the normal operation state based on history and current information. The frame of PHM technology is shown in Figure 3.

As Figure 3 shows, PHM technology makes use of sensors to collect characteristic parameters related to system properties, and combines them with historical data and model parameters. With the help of an algorithm and a model, detection and diagnosis/prediction of defects can be conducted, and manage decision focusing on working state will be made further more. That is to say, whether defect alarms are needed can be judged through this method. As well, residual life, degradation degree and the probability of finishing a mission can be predicted by taking advantage of PHM technology.

The formation of process defects results from inappropriate control of relevant factors during the manufacturing process. This study uses the idea and method of PHM as reference. The concept of failure diagnosis and prognosis is utilized to detect process defects. The core of manufacturing defect detection, based on PHM, is detecting significant process factors to attain the goal of diagnosing and prognosticating process defects. Furthermore, the generation of process defects can be minimized.

The relationship model of process defects influence, which is studied in this paper, is a quantitative description of defect characterization parameters and process-related parameters. The model is the main basis for determining detection parameters and process defect criteria. The process defects detection method based on PHM involves collecting process control parameters related to the manufacture by sensors, comparing the data with the defect criteria, making a judgment about whether defective products will be produced, and taking the corresponding production decisions.

On-line detection technology has wide application in production. Various sensors are utilized to perform real time detection of products’ parameters and obtain relative data. These data are compared with preset parameters in order to make process decisions [11]. Dating back to the early 1940s, manufacturing process on-line detection was applied in industrial production in developed countries and regions, such as Europe and America. Nowadays, with the boom of electronic, sensors, computer and information process technology, process defect detection has already developed as a comprehensive technology referring to the disciplines and technology of automation, electronic, computer and information processing. The development foundation of on-line detection technology is the development of sensor technology, which allows efficient monitoring to be realized. At present, the main development directions of sensor technology are: (a) research on new sensors; (b) development of new materials; (c) integrated sensors; (d) intelligent sensors; (e) wireless sensor nets, etc. [12].

The current manufacturing process defect detection of method involves nondestructive testing technology. Relative standards such as ASME (USA), EN (Europe), JIS (Japan) and ISO were set up in the 1990s, and have been supplemented and updated constantly [13]. The development directions of defect nondestructive testing technology are positive and automatic nondestructive evaluation. For instance, surface defects automatic detection based on image recognition technology such as texture analysis has been successfully applied in the textile field and forging [14,15,16,17]. Meanwell, laser technology has been used to automatically detect weld quality [18] and machine vision pattern recognition technology has been the basis of cigarette packaging automatic detection and classification [19]. With the extension of the non-destructive testing application field to new areas, new problems appear continuously. Giordano in Italy successfully monitored the curing degree and residual stress of cementing processes. They used twisted fiber and Bragg grating sensors to detect the changes in epoxy resin density during the curing stage, but the expensive instruments and complex algorithm make its wide application difficult [20]. There are no perfect detection methods for non-destructive testing for composite material cementing so far. Zheng and others used acoustic detection in their research, but only some preliminary results were obtained. On the other hand, their achievements are too targeted to put into widespread use [21,22,23].

Our research is on the latent defects of ferrite phase shifter insufficient bonding strength, and studies the properties of the resin cementing process. At the same time, a quantitative relationship model between bonding strength and curing temperature, time and pressure is established based on PHM theory and the Avrami formula. Furthermore, key testing parameters are confirmed and a multi-sensor on-line nondestructive testing method is developed. The problem of insufficient bonding strength is solved to a certain extent.

## 2. Cause Analysis of Cementing Process Defects

**Figure 4:**
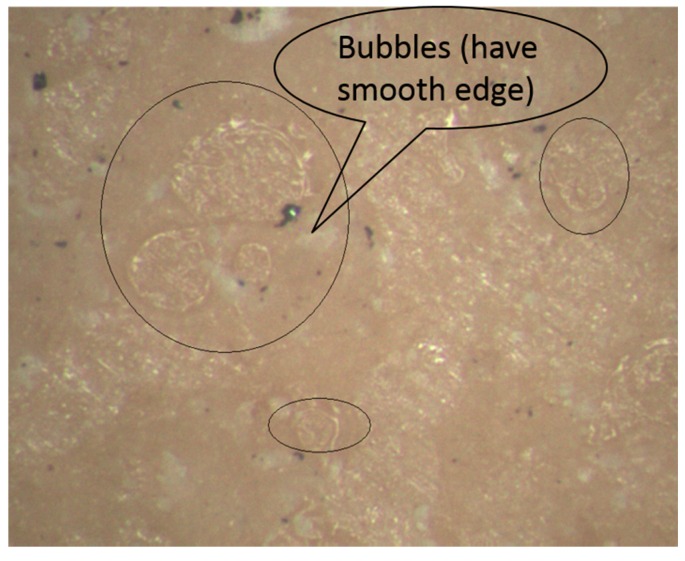
Cementing process defects.

The ferrite phase shifter cementing process is a procedure that bonds microwave dielectric rods and ferrite bars in order while meeting specific requirements of size and strength. The size fluctuation of cementing products is extremely small (±0.03 mm). Relatively, the cementing strength has a greater fluctuation. Hereby, cementing strength is determined to be the defect characterization parameters of cementing process. An optical stereomicroscope is used to observe the crack section of failed parts and qualified parts. Figure 4 illustrates the crack section image of a failed part.

It can be seen from Figure 4 that there obvious bubbles exist on the fracture surface of the failed part. The edge of the bubbles is smooth and little holes appear. That is the reason for the insufficient bonding strength.

Without considering factors such as device failure and the operational environment, cementing strength is related to cementing materials, joint dimension, curing process, etc. They can be summarized as follows:

(1) Cementing materials: different adhesives present different properties. The main factor that has an impact on cementing strength is the chemical groups in the adhesive.
(2) Thickness of cementing coating: the relationship curve of thickness and strength has a single-peak. Over-thick or over-thin cementing coatings will individually lead to a decline of strength.
(3) Treatment for joint surface: adhesive bonding is formed by mechanical, chemical, and adsorption factors. After surface treatment, variations of structure, morphology, chemical constituents and form of organization will occur on the joint surface. All changes of surface condition will have a great influence on the cementing properties.
(4) Curing time and temperature: curing time and temperature are related to cementing materials. If the curing temperature is too low, the molecular chain of adhesive move difficultly. This results in low crosslinking density and incomplete curing reactions, and then influences the cementing strength. The curing time must be prolonged in order to have a complete curing reaction. On the contrary, there is an adhesive loss when the temperature is high. It also causes a decline of cementing strength.
(5) Curing pressure: the role of curing pressure is to ensure that there is a close contact between adhesive and adherends. It is beneficial to expel gas and this makes the cementing layer uniform and compact. Bonding strength increases with the rise of curing pressure within a certain range, but a greater pressure leads to an increase of excessive glue, which may cause incomplete cementing coating and poor strength.
(6) Dimension and shape of joints: whether the joint design is reasonable directly affects the cementing quality. Good designs should avoid stripping, bending and impact loads [24,25,26,27,28,29].

Some of the factors described above as having an effect on the cementing process belong to the category of process design, such as treatments for the joint surface, materials, dimensions and shape. In the process of product manufacturing, these kinds of basic factors are determined, and will not change. Except for process design factors, cementing strength is mainly related to the following process control factors: thickness of adhesive, curing temperature, curing time and curing pressure. Curing pressure has an impact on the thickness of the adhesive, but the thickness is only about 0.02 mm, so the fluctuations of adhesive thickness are so small that its influence can be ignored, so the main process parameters related to cementing strength in the ferrite phase shifter cementing process are curing temperature, curing time and curing pressure.

## 3. Relation Model of Cementing Process Parameters

### 3.1. The Avrami Formula

The main composition of adhesive used in ferrite phase shifter cementing process is modified epoxy resin. The cementation procedure of the cementing process is a curing process of adhesive at high temperature.

(1) Role of process parameters

According to the curing theory of thermosetting resin and cementing process, the main process parameters related to cementing strength (curing temperature, curing time and curing pressure) play significant roles, which are as follows:

(a) Curing temperature: it mainly affects the curing temperature of the adhesive material, that is the curing rate of the thermosetting resin. On the one hand, a low temperature will slow down the curing process. The bonding performance will be affected if the curing time of the specification is still adopted. On the other hand, a high temperature will make the solvent of the adhesive volatilize earlier, influencing its viscosity. Meanwhile, it will also result in aging of the cured resin.
(b) Curing time: it mainly affects the degree of curing of thermosetting resin. Curing time and temperature depend on each other within a certain range. Increasing the temperature (that is speed of the curing rate), can shorten the curing time.
(c) Curing pressure: the effect of curing pressure is expelling bubbles in the adhesive and volatile solvent, as while as controlling the thickness of the cementing coating. Over-stress can cause a serious lack of glue. Inversely, insufficient pressure may make the cementing layer loose, thick, uneven and have large quantities of bubbles.

From the above, curing temperature and curing time affect the curing degree collectively. Cementing strength is related to curing degree directly. Curing stress influences the factors such as internal uniformity and bubbles, and further causes poor bonding strength.

(2) The Avrami formula of the thermosetting resin’s curing process

According to the mentioned above, curing degree is one of the most important factors affecting the bonding strength in cementing processes. The phase-change theory of Avrami is proved to describe well the isothermal curing of thermosetting resins as while as the curing process with uniform velocity and temperature variation. The Avrami formula [30,31,32] explains the dynamic process of polymer crystallization. Some researchers [33,34] make use of the Avrami equation to simulate the curing process of resin curing systems. The relative curing degree *α* corresponds to relative crystallinity. Curing time (*t* − *t_g_*) after gelation time *t_g_* corresponds to crystallization time. In that way, the dynamic equation describing epoxy resin curing process can be expressed as:

(1) $$\alpha =1-exp\left[-k{\left(t-t_{g}\right)}^{n}\right]$$

where *α* is relative curing degree after the gel point, *k* is a constant representing curing rate after the gel point, *n* is the Avrami index characterizing the reaction mechanism, *t* is the curing time, and *t_g_* is the gelation time.

The constant *k* in Equation (1) has a temperature dependency. Meanwhile, curing is a thermal activation process. The equation between *k* and curing temperature is as follows [34]:

(2) k1n=A exp(−EaRT)

where *k* is the curing constant, *A* the antecedent factor, *E_a_* the activation energy of the curing system after the gel point, *R* the universal gas constant (*R* = 8.31 J∙mol/K), and *n* the Avrami index.

In a word, the cementing process of a ferrite phase shifter has two main factors that affect cementing strength: curing degree and curing pressure. It is known that the two factors are independent of each other. Therefore, the bonding strength of the cementing process can be regarded as a function of curing degree and pressure.

### 3.2. DSC and Process Test of Adhesive Curing Process

(1) DSC test for adhesive curing process

**Figure 5:**
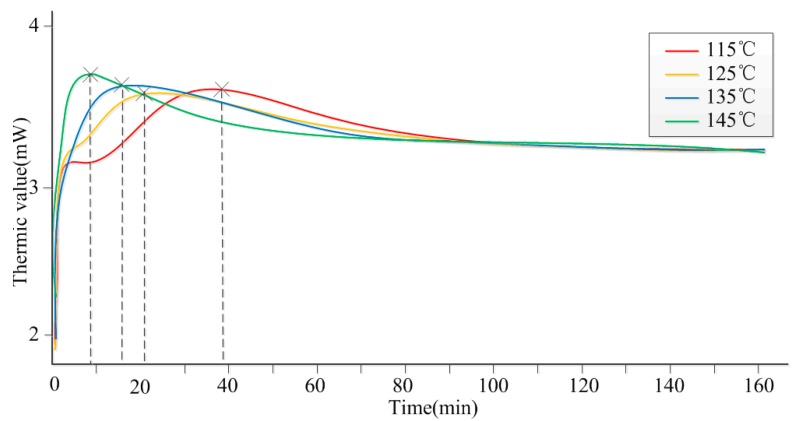
DSC curve of adhesive isothermal curing.

In order to determine the dynamic parameters of the curing process, and the quantitative relationship between curing degree and curing temperature, curing time, a DSC test was conducted at four temperature levels ranging from 115 °C to 145 °C. This method is one of the most mature tools to research the process kinetics of thermosetting resins curing processes. The DSC curve shows the correlativity between samples’ exothermic/endothermic value and time. In our case, the adhesive curing process in an exothermic reaction, and its DSC curve is shown in Figure 5.

It is shown in Figure 5 that the time for the resin adhesive to display its maximum heat release is different under different temperatures. Specifically, it is 40 min at 115 °C, 20 min at 125 °C, 15 min at 135 °C and 10 min at 145 °C. When the heat emission achieves its maximum, the resin adhesive starts to curing. That is to say, without considering curing pressure, curing time has a negative correlation with temperature. It is known that gas and water in the resin will release easier at higher temperature. Better bonding strength as the output will be obtained. However, temperature is not the only influencing factor, as curing pressure needs to be considered, too. Therefore, tests concerning temperature and pressure were conducted to help with the research.

(2) Process test for cementing process

**Figure 6:**
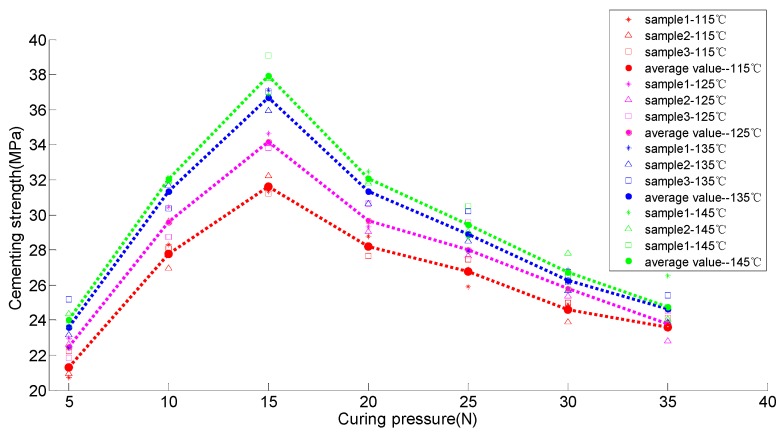
The test data of ferrite phase shifter cementing process.

For the sake of establishing the relation between cementing strength and curing degree and curing time, 28 group tests were conducted. There were four different temperatures (115, 125, 135 and 145 °C). Under each temperature there were seven groups, corresponding to seven curing pressures (5, 10, 15, 20, 25, 30 and 35 N). Three samples were extracted from each group to make comparisons. Due to different curing time under different temperatures, we set 2 h so that every sample could cure. The process test combined engineering experience and the DSC analysis results. In addition, the limiting expenses and time were also considered. The test scheme and data records are shown in Table 1 and Figure 6.

It can be seen from Table 1 and Figure 6 that at the same temperature, the bonding strength increased when the pressure changed from 5 N to 15 N. On the contrary, the bonding strength decreased when the pressure increased continually, from 15 N to 35 N. This means that the curing pressure had a larger influence on the bonding strength, and the best result occurred at 15 N. On the other hand, under the same pressure, bonding strength had a positive correlation with temperature. Bonding strength at 135 °C was much better than that at 115 °C and 125 °C. Measured results at 135 °C and 145 °C were close. This provided a key basis for confirming the detection parameters.

### 3.3. The Establishment of Cementing Process Parameters’ Relation Model

(1) Adhesive curing process kinetic parameters

**Figure 7:**
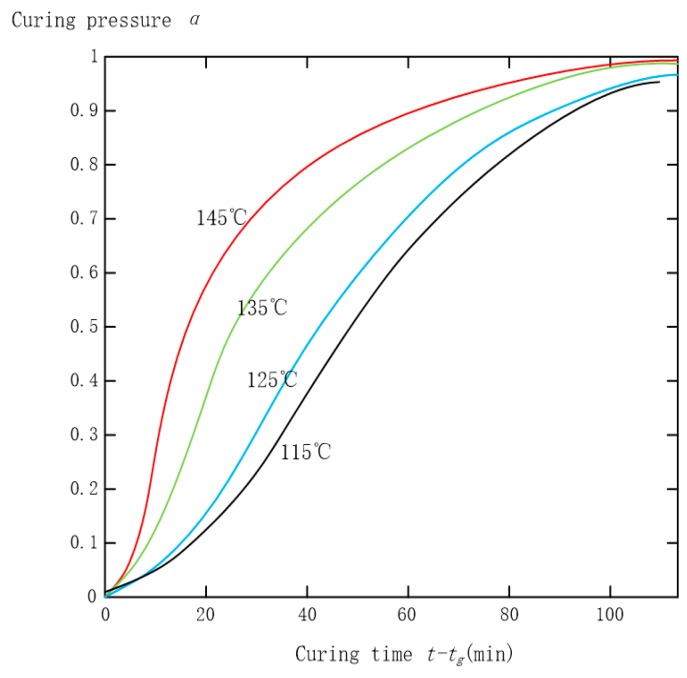
Relationship between curing degree and curing time of adhesive isothermal curing process.

According to the DSC test data illustrated in Figure 5, a relationship between curing degree and curing time of the adhesive isothermal curing process can be obtained through a conversion calculation, depicted in Figure 7. Obviously, the isothermal curing process is consistent with the Avrami formula.

The Equation (3) is derived from Equation (1):

ln[−ln(1 − *α*)] = ln*k* + *n* ln(*t* − *t_g_*) (3)

**Figure 8:**
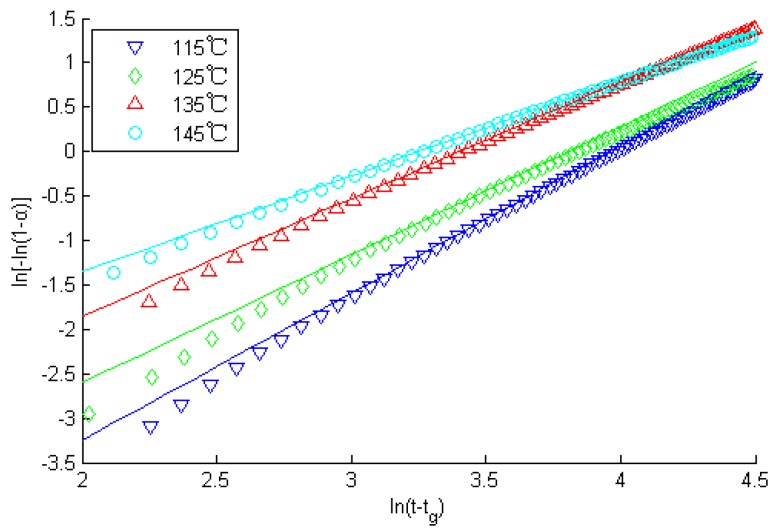
Avrami fitting of the adhesive isothermal curing process under different temperatures.

It is known that ln[−ln(1 − *α*)] and ln(*t − t_g_*) are linearly dependent. A graph (Figure 8) can be drawn with ln(*t − t_g_*) as the abscissa and ln[−ln(1 − *α*)] as the ordinate. Thus, the values of the Avrami index *n* and Avrami rate *k* are available; *α* is the relative curing degree after the gel point, and *t* the curing time, *t_g_* the gelation time. They are listed in Table 2.

The constant *k* has a temperature dependency. Based on Equation (2), the relationship between *k* and curing temperature *T* can be represented as follows:

(4) $$\frac{1}{n}\ln k=-\frac{E_{a}}{RT}+\ln A$$

**Figure 9:**
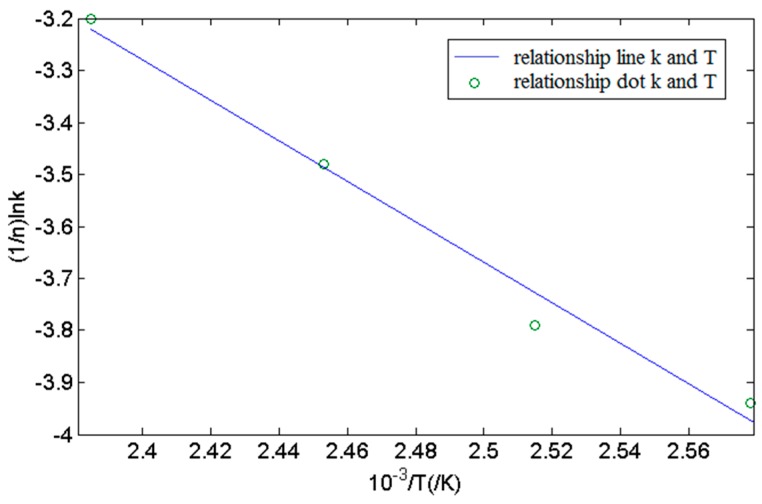
The relationship between Avrami rate k and the curing temperature T.

It is clear that (*1/n*)∙ln*k* is linear to *1/T*. ln(*t − t_g_*) is made to be the abscissa and ln[−ln(1 − *α*)] the ordinate, and Figure 9 is accomplished. Values of activation energy *E_a_* of curing system reaction and antecedent factor *A* are available. They are shown in Table 2 too.

From Figure 7 and Figure 8, Table 2, we can know the fitting graphic that uses test data complied with the principle of the Avrami formula. In the meantime, relative parameters in the formula were ensured under different temperatures, such as gelation time *t_g_*, Avrami index *n*, Avrami rate constant *k*, system’s activation energy *E_a_*, antecedent factor *A*, etc. As mentioned earlier, it proved that the higher the temperature, the shorter the curing time.

(2) Relationship of curing degree and bonding strength

**Figure 10:**
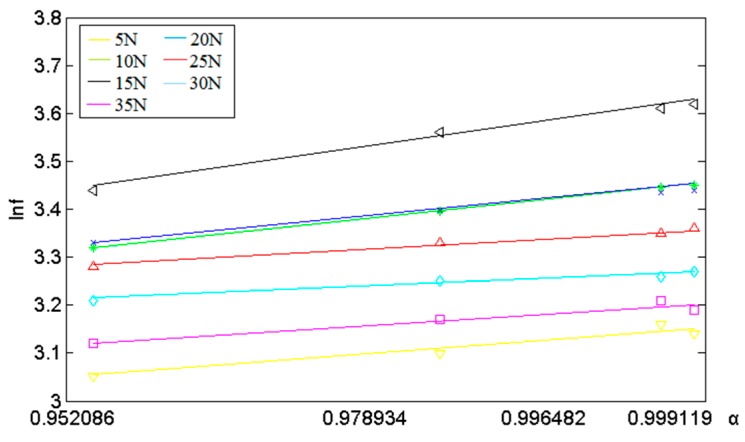
Curing strength and curing degree under different curing pressure.

From the results of the DSC test, it can be seen that different curing temperatures and times correspond to different curing degrees *α*. The values are: (a) 115 °C, 2 h: 0.9521; (b) 125 °C, 2 h: 0.9789; (c) 135 °C, 2 h: 0.9965; (d) 145 °C, 2 h: 0.9992, respectively. Data shown in Table 3 are used to plot a graph (Figure 10) expressing the curve of ln*f* (*f* represents boing strength and its unit is MPa) and *α*.

It can be inferred from the graph shown in Figure 10 above that ln*f* and *α* have a good linear relationship. Meanwhile, there is a bunch of approximate parallel lines under the conditions having different curing pressure. Therefore, it can be concluded that bonding strength *f* and curing degree *α* has the following relation:

(5) $$f=f_{1}\left(\alpha \right)f_{2}\left(N\right)=B_{1}e^{b\alpha }f_{2}\left(N\right)$$

where *f*_1_(*α*) is the impact that curing degree had on cementing strength; *f*_2_(*N*) the influence that curing pressure made on cementing strength; *B_1_* and *b* the adjustment coefficients.

(3) Relationship of curing pressure and bonding strength

**Figure 11:**
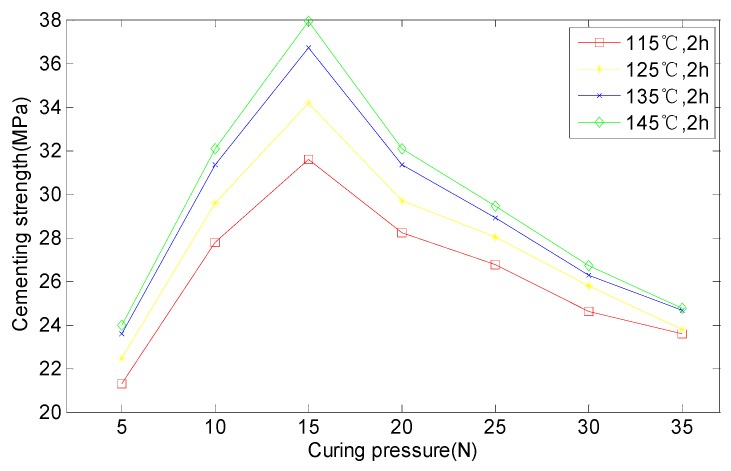
Relation between *f* and *N* under different curing degree.

We make *f* the ordinate and *N* the abscissa, then Figure 11 can be drawn according to data in Table 4.

From Figure 11, we can see that the real curve is largely in line with the ideal curve drawn according to Equation (5). It can be inferred that cementing strength *f* and curing pressure *N* have the following relation:

(6) $$f=f_{1}\left(\alpha \right)f_{2}\left(N\right)=f_{1}\left(\alpha \right)B_{2}Ne^{-cN}$$

where *f*_1_(*α*) is the impact that curing degree had on cementing strength; *f*_2_(*N*) the influence that curing pressure made on cementing strength; *B_2_* and *c* are also the adjustment coefficients.

(4) Relation model of cementing process parameters

Based on the analysis of the above results, a quantitative relationship between cementing strength and curing temperature, curing time, curing pressure during the cementing process is acquired:

(7) {f=BN exp(bα−cN)α=1−exp[−k(t−tg)n]k1n=A exp(−EaRT)

Here, *f* is the cementing strength (MPa); *T* the curing temperature (K); *t* the curing time (min); *N* the curing pressure (N); *α* the curing degree. *k* and *n* are the rate constant and Avrami index, respectively; *t_g_* the gelation time (min); *E_a_* the activation energy of curing reaction (J∙mol); R the universal gas constant (*R* = 8.31 J∙mol/K). *A, B, b* and *c* are all adjusted factor.

According to Equation (7), it is clear that there is a linear relationship between ln(*f/N*) and curing degree *α*, and curing pressure *N*. It is shown as Equation (8). We used the data in Table 1 and through binary linear regression analysis, the parameters in Equation (7) relative to the model can be obtained. The multiple correlation coefficient of regression analysis is 0.9908, which means that the assumptive model is reasonable:

ln(*f/N*) = *bα* − *cN* + ln*B* (8)

Here, *f* is cementing strength (MPa); *N* the curing pressure (N); *α* is the curing degree; *B*, *b* and *c* are all adjusted factor. Combined with the kinetic parameters related to adhesive curing process, the final parameters of the model are listed in Table 3.

The relationship model of cementing process parameters was established on the basis of the Avrami formula. All the values of the relative parameters are listed in Table 3. We obtained adjusted factors *B*, *b* and *c*. These three factors had a direct impact on the relationship of curing temperature, curing pressure and curing time.

## 4. The Decision Criterion of Cementing Process Defect Detection Parameters

On the basis of Equation (7), sensitivity analysis of the process parameters can be conduced. Equation (9) is the sensitivity calculation formula for curing temperature, curing time, curing pressure and cementing strength:

(9) $$\{\begin{array}{c} \frac{\partial f}{\partial N}=\left(1-cN\right)B\;exp\left(b\alpha -cN\right)\\ \frac{\partial f}{\partial t}=\frac{\partial f}{\partial \alpha }\frac{\partial \alpha }{\partial t}=BbknN{\left(t-t_{g}\right)}^{n-1}exp\left[b\alpha -cN-k{\left(t-t_{g}\right)}^{n}\right]\\ \frac{\partial f}{\partial T}=\frac{\partial f}{\partial \alpha }\frac{\partial \alpha }{\partial k}\frac{\partial k}{\partial T}=BbnA^{n}N\frac{Ea}{RT^{2}}{\left(t-t_{g}\right)}^{n}exp\left[b\alpha -cN-k{\left(t-t_{g}\right)}^{n}-\frac{nEa}{RT}\right] \end{array}$$

In line with Equation (9) and Table 4, it can be known that when the curing pressure is 15.7 N, *əf*/*əN* ≈ 0. This means that the maximum value of cementing pressure is achieved when the curing pressure is 15.7 N. Basing on process tests and engineering requirements, the optimal process condition of the cementing process is: curing temperature: 135 °C; curing time: 2 h; curing pressure: 15 N~16 N. The sensitivity at the very process condition is displayed in Table 4.

From Table 4, some conclusions can be drawn. In a certain range of process conditions, pressure and temperature have a stronger influence on cementing strength. There is a positive correlation between temperature, time and strength. In other words, within a certain scope, both moderately increasing the curing temperature and curing time can improve the cementing strength. To sum up, the defect detection parameters of cementing process are curing pressure and curing temperature.

## 5. Case of Cementing Process Defect Detection and Accessment

### 5.1. The Establishment of the Multi-Sensor PHM System Detection Scheme

**Figure 12:**
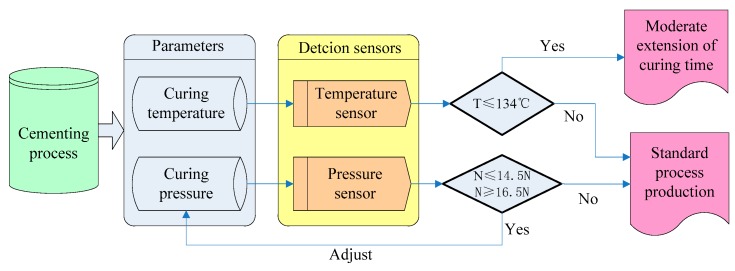
Cementing process defect detection scheme design.

We have already known that the defect detection parameters are curing pressure and curing temperature. A temperature and pressure multi-sensor system is established based on the actual situation and analysis resultss above. Functions of the system are detecting, diagnosing and controlling cementing process defects on-line. Figure 12 is the scheme design diagram.

In Figure 11, in view of the ferrite phase shifter cementing process, detection parameters (curing temperature and curing pressure) of potential defects were ensured. According to the PHM model shown in Figure 3, temperature sensors were used in on-line detection of the curing temperature, while pressure sensors were used in the curing pressure’s on-line detection. Based on the on-line detection criteria confirmed from Table 3 and Table 4, we can be sure that the normal conditions are a temperature higher than 134 °C and a pressure between 14.5 N and 16.5 N. When the temperature is lower than 134 °C and the pressure is lower than 14.5 N or higher than 16.5 N, the cementing process is in an abnormal state. If the temperature does not meet the criterion, curing time should be prolonged. If it is a result of pressure, it should be adjusted in time.

**Figure 13:**
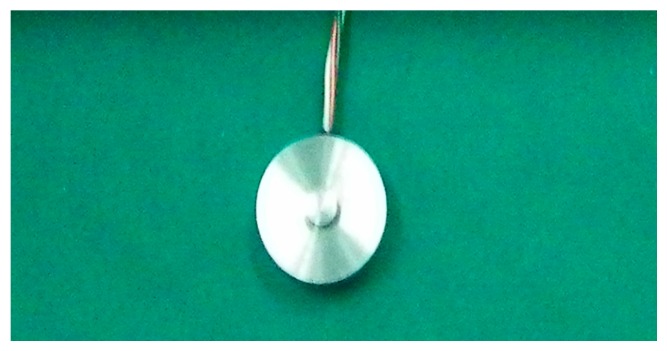
Pressure sensor.

**Figure 14:**
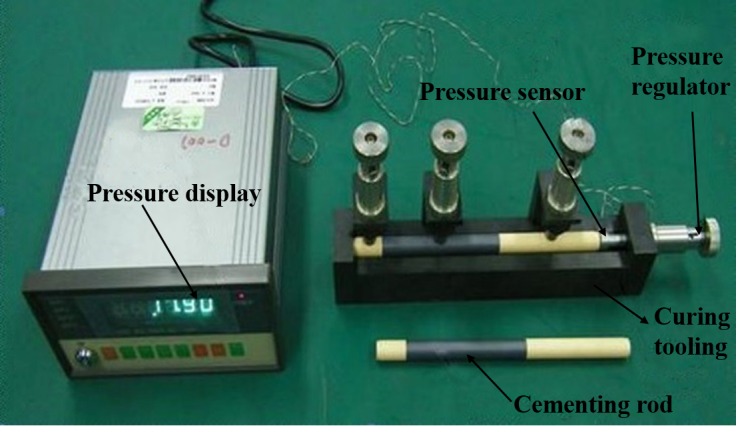
Set mode of detection sensor.

Regarding the temperature sensor setting, because the curing process occurred in an incubator which has a temperature sensor and display, the curing temperature can be directly controlled through the temperature detection and display of the incubator. The pressure sensor setting is shown in Figure 13 and Figure 14.

Figure 14 is the sensor used during the cementing process. Figure 14 shows the way the pressure sensor works. It was put on the right surface of the ferrite phase shifter, and had a connection to a pressure adjusting device. The adjusting device adjusted the value of thevcuring pressure. The value could be read through the pressure sensor, so we could judge whether it exceeded the criterion.

### 5.2. Accessment

The measurement of defect detection and control during the cementing process was applied in the actual production of a ferrite phase shifter. We counted up the bonding strength data before using the detecting method (the specific data is too abundant so is not listed). In total, there are five groups, 151 samples, and the percent of qualified products was nearly 85%. In contrast, after making use of the on-line detection method, the percent of qualified products was above 95% (eight groups, 242 samples). The qualification rate of the ferrite phase shifters was increased by 11.8%.

**Figure 15:**
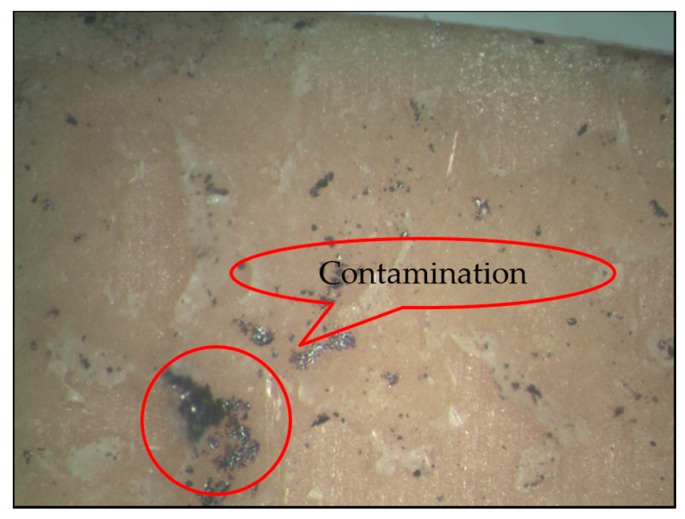
Cementing material contamination.

After the application of the detection method, there were 12 sub-quality products among the 242 samples. During the cementing process, no problems with these 12 products had been found by the on-line detection method. Analyzing all 12 products, we found that eight products had contamination on the cementing material surface, shown in Figure 15, so the problem was not a result of abnormal curing temperature or pressure. Two products had material cracks. Again it was not a cementing process problem. The other two had bubbles on the cementing surface. These were cementing process problems that our method did not detect.

According to the 85% qualification rate without using this method, there might be 36 (242 × 15%) unqualified samples among total 242 sample. Except for the 10 unqualified samples which failure was not caused by cementing process problems, there were 26 samples that might have cementing process problem. Among the 26 samples, two were undetected, so the probability that the on-line detection method could find and rectify the potential defects was 92.3% (24/26).

**Figure 16:**
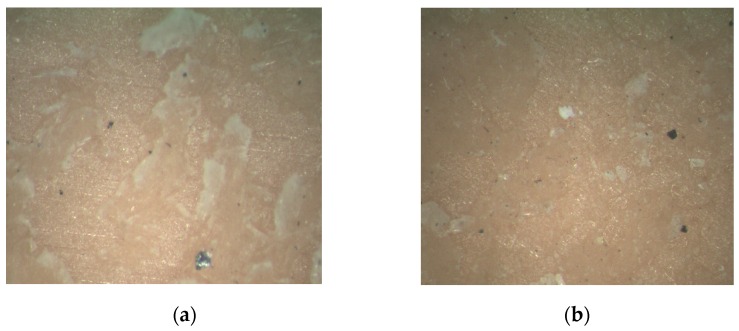
Transverse morphology after applying the detection and control measures: (**a**) One face of the cementing surface; (**b**) Another face of the cementing surface.

On the other hand, after the control measures were applied, ten qualified products were chosen for analysis and verification. The morphology of a transverse section showed that there were only a small amount of air bubbles, and their area was small, as shown in Figure 16.

From what has been discussed above, it can be inferred from the above analysis that the method for detection and measurement of cementing process defects put forward in this paper is effective. A deficiency of this method is that material defects such as ferrite material defects (cracks, uneven surface, etc.) or resin adhesive material defects (pollution, aging, etc.) cannot be detected by the on-line detection method.

With the development of related technology, new methods aimed at defects hard to detect will appear, but cost, complexity of algorithma and feasibility to implement are factors that need to be considered. As for our the object of study, cementing material defects may exist, but it is difficult to use conventional sensors to observe them. According to these undetected defects, an indirect on-line non-destructive testing method will be researched next.

## 6. Conclusions

The ferrite phase shifter manufacturing process is the application analysis object of this paper. The analysis of its cementing process defects was completed and a quantitative relation model is finally established. The model involves curing time, temperature, pressure and cementing strength. A method combining the Avrami formula and test data is applied. We determined the process parameters which have a stronger effect on cementing strength, by sensitivity analysis of curing time, curing temperature and curing pressure. On-line detection of pressure and temperature multi-sensor PHM system is applied to ensure the detection method for cementing process defects. In the end, the qualification rate of the cementing process is increased. This is of great significance for improving the reliability of the ferrite phase shifter manufacturing process. In future works, an indirect on-line non-destructive testing method for undetected defects will be studied.

## Figures and Tables

**Table 1:** The test scheme and data records of the ferrite phase shifter cementing process.

	Process Factors	Curing Temperature (°C)	Curing Time (h)	Curing Pressure (N)	Cementing Strength (MPa)			
Groups					Sample 1	Sample 2	Sample 3	Average Value
1		115	2	5	20.7	20.96	22.24	21.3
2				10	28.31	26.96	28.1	27.79
3				15	31.32	32.24	31.24	31.6
4				20	28.78	28.18	27.64	28.2
5				25	25.92	26.86	27.47	26.75
6				30	24.91	23.9	25.0	24.6
7				35	23.8	22.8	24.14	23.58
8		125	2	5	22.97	22.6	21.84	22.47
9				10	30.43	29.63	28.74	29.6
10				15	34.63	34.05	33.83	34.17.
11				20	29.31	29.05	30.64	29.67
12				25	26.89	27.64	29.54	28.02
13				30	26.18	25.37	—	25.78
14				35	23.94	22.8	24.53	23.76
15		135	2	5	22.46	23.15	25.18	23.6
16				10	32.03	31.67	30.36	31.35
17				15	37.1	35.96	37.04	36.7
18				20	32.03	30.64	—	31.34
19				25	27.97	28.52	30.2	28.9
20				30	26.89	25.67	26.18	26.25
21				35	24.66	23.86	25.4	24.64
22		145	2	5	23.94	24.37	23.7	24.0
23				10	32.17	31.92	—	32.05
24				15	36.86	37.78	39.08	37.91
25				20	32.46	31.8	31.92	32.06
26				25	28.72	29.06	30.48	29.42
27				30	25.84	27.8	26.55	26.73
28				35	26.53	24.05	23.67	24.75

“—”: Ferrite bar or microwave dielectric rod was fractured, and the data was removed.

**Table 2:** Kinetics parameters of the adhesive isothermal curing process.

Curing Temperature		Gelation Time *t_g_* (min)	Avrami Index *n*	Avrami Rate Constant *k*	System’s Activation Energy *E_a_* (kJ∙mol)	Antecedent Factor *A*
(°C)	(K)					
115	388	12.66	1.665	0.001417	34.17	746.2
125	398	4.38	1.46	0.003966		
135	408	2.85	1.258	0.01287		
145	418	1.63	1.104	0.02919		

**Table 3:** Relationship model parameters of ferrite phase shifter cementing process.

Curing Temperature (K)	Gelation Time *t_g_* (min)	Avrami Index *n*	Avrami Rate Constant *k*	Activation Energy of Curing Reaction *E_a_* (kJ·mol)	Adjusted Factor			
					*A*	*B* (MPa/N)	*b*	*c* (1/N)
388	12.66	1.665	0.001417	34.17	746.2	0.6018	2.3195	0.0635
398	4.38	1.46	0.003966					
408	2.85	1.258	0.01287					
418	1.63	1.104	0.02919					

**Table 4:** Process parameter sensitivity of ferrite phase shifter cementing process.

Process Condition	Process Parameter Sensitivity to the Cementing Strength		
	Curing Pressure	Curing Time	Curing Temperature
	$\frac{\partial f}{\partial N}$ (MPa/N)	$\frac{\partial f}{\partial t}$ (MPa/min)	$\frac{\partial f}{\partial T}$ (MPa/K)
135 °C, 2 h, 15 N	0.1112	0.02582	0.0742
135 °C, 2 h, 16 N	−0.0352	0.02585	0.0743
